# ASO Author Reflections: Neoadjuvant Chemotherapy in cT2N0M0 Gastric Cancer; Time to Revisit Current Recommendations?

**DOI:** 10.1245/s10434-024-15688-w

**Published:** 2024-07-04

**Authors:** Francesco Abboretti, Markus Schäfer, Caroline Gronnier, Styliani Mantziari

**Affiliations:** 1 Department of Visceral Surgery, Lausanne University Hospital CHUV, Lausanne, Switzerland; 2 Faculty of Biology and Medicine, University of Lausanne UNIL, Lausanne, Switzerland; 3 Eso-Gastric Surgery Unit, Department of Digestive Surgery, Magellan Center, Bordeaux University Hospital, Pessac, France; 4 Faculty of Medicine, Bordeaux Ségalen University, Bordeaux, France

## Past

The optimal management of cT2N0M0 gastric adenocarcinoma is still a matter of debate. Despite guidelines suggesting neoadjuvant chemotherapy for cT2N0M0 patients,^1,2^ clinical practice remains controversial, as landmark studies such as the recent FLOT4 trial have not specifically addressed this subgroup.^3^ Similarly to cT2N0 esophageal cancer, where upfront surgery is the recommended treatment strategy,^4^ previous retrospective data have suggested a lack of benefit of systemic treatment in cT2N0M0 gastric cancer.^5^

## Present

Due to the low level of evidence, treatment protocols for cT2N0M0 gastric adenocarcinoma are often dependent on local institutional preferences, leading to large heterogeneity in clinical practice. A recent large-scale multicentric cohort study aimed to address this issue, assessing the impact of neoadjuvant chemotherapy on survival and recurrence outcomes of cT2N0M0 patients.^6^ In this series, preoperative chemotherapy was more often offered to younger, more “fit” cT2N0M0 patients with proximal tumors. However, no increased R0 resection rates, or improved overall and recurrence-free survival, were observed.^6^ These findings underline the prevailing assumptions that this group of patients does not necessarily benefit from systemic treatment. Aggressive (undifferentiated) histology, extensive submucosal infiltration, or potentially compromised surgical margins might represent specific risk factors that could be used to identify subgroups of patients with a potential benefit from neoadjuvant chemotherapy.

## Future

A better understanding of gastric adenocarcinoma development, gastric cancer subtypes, individual risk factors, and pathophysiology of recurrence is mandatory to define improved treatments. The biomolecular classification of gastric cancer into four distinct subtypes (EBV-related, microsatellite-instable, genomically stable, chromosomally instable)^7^ has had little impact in clinical practice thus far, highlighting the challenges of translating basic research findings into clinical treatment concepts. Nevertheless, exploring the gastric adenocarcinoma microbiome and molecular characteristics remains a promising field of interest,^8,9^ suggesting potential pathways for future breakthroughs in treatment and management.
